# Supplementary material for: Haze Occurrence Caused by High Gas-to-Particle Conversion in Moisture Air under Low Pollutant Emission in a Megacity of China
Source: Int J Environ Res Public Health. 2022 May 25;19(11):6405. doi: 10.3390/ijerph19116405 (PMC9179953; doi:10.3390/ijerph19116405)
Supplement: Supplementary file 1 [file ijerph-19-06405-s001.zip › ijerph-1695979-supplementary.pdf]

Supplementary Materials:

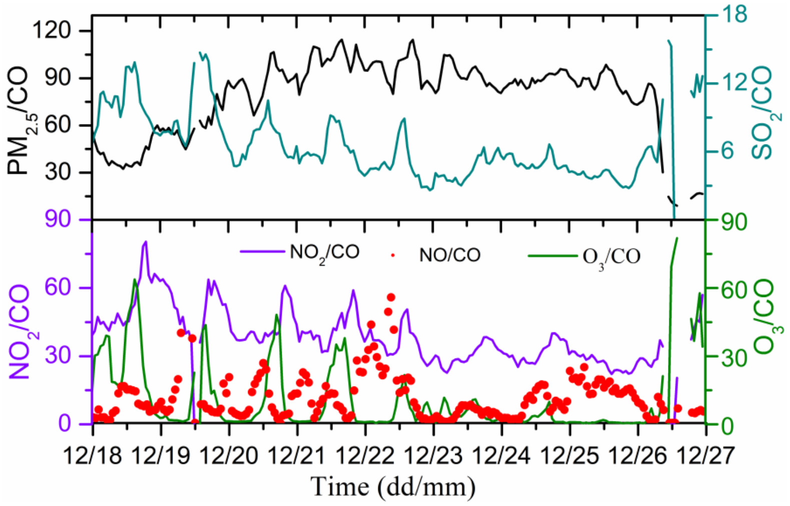

Figure S1. Time series of $PM_{2.5}$ and gaseous pollutants (NO, NO_2, SO_2, CO and O_3) in the three periods.

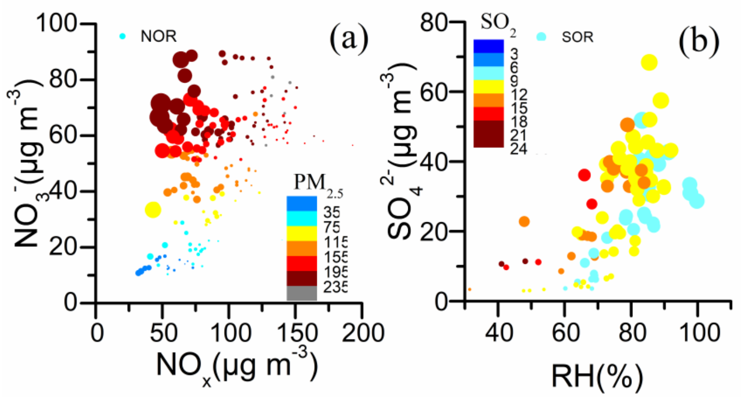

Figure S2. Correlations between NO_3^- and NO_x, SO_4^{2-} and RH. Symbols in (a) are scaled by NOR, and colored by $PM_{2.5}$ concentration; Symbols in (b) are scaled by SOR concentration, and colored by SO_2.

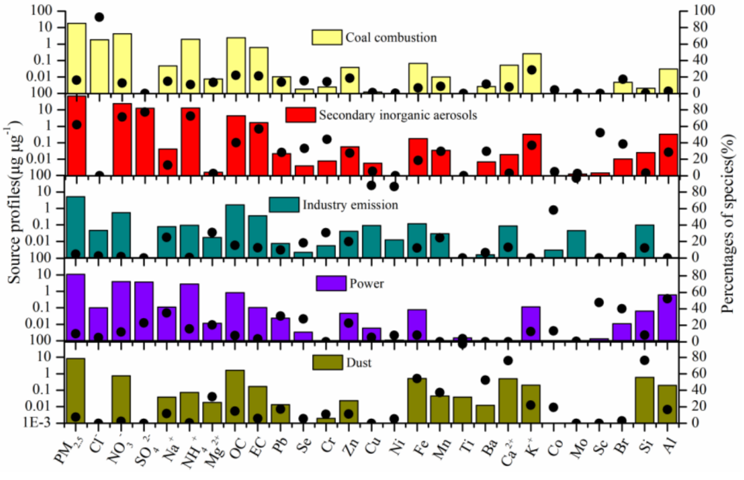

Figure S3. Profiles of four sources profiles (bars) resolved from the PMF model (in units of μg μg^{-1}) and contribution percentages (black dots) from each source factor.
